# Implementation framework for chronic disease intervention effectiveness in Māori and other indigenous communities

**DOI:** 10.1186/s12992-017-0295-8

**Published:** 2017-09-05

**Authors:** John Oetzel, Nina Scott, Maui Hudson, Bridgette Masters-Awatere, Moana Rarere, Jeff Foote, Angela Beaton, Terry Ehau

**Affiliations:** 1 University of Waikato, Private Bag 3105, Hamilton, 3240 New Zealand; 2 Waikato District Health Board, Pembroke Street, Private Bag 3200, Hamilton, 3240 New Zealand; 3 The Institute of Environmental Science and Research, 34 Kenepuru Drive, PO Box 50348, Porirua, 5240 New Zealand; 4 Waikato Institute of Technology, Private Bag 3036, Waikato Mail Centre, Hamilton, 3240 New Zealand

**Keywords:** Kaupapa Māori, Community-engaged research, Systems thinking, Culture-centeredness, Integrated knowledge translation, Implementation science

## Abstract

**Background:**

About 40% of all health burden in New Zealand is due to cancer, cardiovascular disease, and type 2 diabetes/obesity. Outcomes for Māori (indigenous people) are significantly worse than non-Maori; these inequities mirror those found in indigenous communities elsewhere. Evidence-based interventions with established efficacy may not be effective in indigenous communities without addressing specific implementation challenges. We present an implementation framework for interventions to prevent and treat chronic conditions for Māori and other indigenous communities.

**Theoretical framework:**

The He Pikinga Waiora Implementation Framework has indigenous self-determination at its core and consists of four elements: cultural-centeredness, community engagement, systems thinking, and integrated knowledge translation. All elements have conceptual fit with Kaupapa Māori aspirations (i.e., indigenous knowledge creation, theorizing, and methodology) and all have demonstrated evidence of positive implementation outcomes.

**Applying the framework:**

A coding scheme derived from the Framework was applied to 13 studies of diabetes prevention in indigenous communities in Australia, Canada, New Zealand, and the United States from a systematic review. Cross-tabulations demonstrated that culture-centeredness (*p* = .008) and community engagement (*p* = .009) explained differences in diabetes outcomes and community engagement (*p* = .098) explained difference in blood pressure outcomes.

**Implications and conclusions:**

The He Pikinga Waiora Implementation Framework appears to be well suited to advance implementation science for indigenous communities in general and Māori in particular. The framework has promise as a policy and planning tool to evaluate and design effective interventions for chronic disease prevention in indigenous communities.

## Background

New Zealand faces significant challenges relating to chronic, non-communicable diseases such as diabetes and obesity. Health inequities between Māori (indigenous people of NZ) and non-Māori are particularly concerning [1]. Almost half (47%) of Māori (indigenous people of New Zealand) are obese (Body Mass Index >30) compared to 29% of European/Other New Zealanders [2]. Similarly, 7.2% of Māori have diabetes compared to 5.1% of European/Other New Zealanders [2]. Further, Māori have 1.8 times more health burden (i.e., disability adjusted life years) than non- Māori [3] and the average life expectancy for Māori is nine years less than that of other New Zealanders [4]. These inequities are explained by racism and the unjust distribution of social determinants of health including income, employment, education, housing, and health service inequities in access to, and quality of, health care. This injustice is underpinned by a lack of commitment by the New Zealand government toward meeting its obligations under Te Tiriti o Waitangi—the founding treaty of New Zealand [5–7]. These inequities mirror those found between indigenous and non-indigenous populations in across the globe [8, 9].

In 2012–13 New Zealand Government implemented the National Science Challenges (NSCs) initiative as a mission-led form of research funding to address 11 significant science challenges related to the environment and social/human health [10]. A key goal is to develop innovative scientific approaches with a clear implementation pathway for scalability and larger nationwide impact. The NSCs are guided by the Vision Mātauranga policy which aims “to unlock the innovation potential of Māori knowledge, resources and people to assist New Zealanders to create a better future” (p. 1) [11]. Despite the inclusion of Vision Mātauranga in the NCSs, recent research has critiqued the NSCs for foregrounding scientific knowledge production in the context of neoliberalism, and discussed how Māori researchers reasserted the importance of Māori knowledge production [12].

One of the NSCs is the Healthier Lives Challenge which aims to improve the prevention and treatment of four of New Zealand’s most significant non-communicable diseases: cancer, cardiovascular disease (CVD), diabetes, and obesity. Its mission is “to deliver the right prevention to the right population and the right treatment to the right patient” in order to reduce the burden of these diseases by 25% by 2025 [13]. Within this purpose and mission is a stated goal to reduce health inequities for Māori and other communities by 25% by 2025. This article describes the theoretical foundation for one of the projects in the Healthier Lives Challenge designed to address these inequities: “He Pikinga Waiora [Enhancing Wellbeing]: Making health interventions work for Māori communities.” He Pikinga Waiora references the whakatauki (traditional proverb), *He oranga ngakau, he pikinga waiora,* which refers to the relationship between positive feelings and a sense of self-worth, key aspects of well-being.

Despite a strong international evidence base for a range of interventions that have been shown to improve outcomes for chronic diseases, there has been underwhelming progress made in reducing health inequities [14]. The U.S. National Institutes of Health and other researchers recognize the importance of translational research and implementation science for achieving health equity and has identified issues of context and external validity as central to the problem of the utilization of evidenced-based practices [15–17]. Efficacy studies and randomized controlled trials, which focus on internal validity, are necessary, but frequently do not translate to real-world settings with high variability in culture, context, and levels of acceptance [16, 18–20]. The Canadian Institutes of Health Research (CIHR) have recognised that the creation of new knowledge often does not, on its own, lead to widespread implementation or to positive health outcomes [21]. Translation, dissemination, uptake and implementation are becoming increasingly important to transition innovative health research into health policy and practice and ultimately achieve health equity.

Thus, the challenge of achieving healthier lives for Māori and other indigenous communities needs to move beyond a narrow focus on intervention efficacy to include consideration of effective implementation in specific settings with a focus on prolonging sustainability and facilitating uptake [22]. Our purpose is to describe a theoretical foundation for effective and culturally-appropriate implementation of prevention and treatment interventions for Māori communities. The theoretical framework is introduced and then applied to 13 studies from a systematic review of primary health care interventions for indigenous people with type 2 diabetes [23].

## Theoretical foundation

The implementation framework supports researchers, practitioners and public policy makers to create sustainable and effective intervention pathways to improve health for Māori communities. Given our focus on Māori communities, *Kaupapa Māori* provides theoretical grounding for successful implementation of interventions and reduction of health inequities. A *Kaupapa Māori* approach emphasises local context and self-determination by prioritizing indigenous history, development, and aspirations. Kaupapa Māori initiatives have been associated with improved health outcomes and engagement for Māori [24–27].

**Fig. 1:**
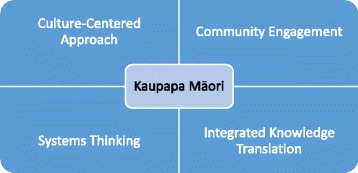
Key elements of implementation framework for Māori communities

The importance of stakeholder knowledge and participation in research, translation, and dissemination of research findings is increasingly acknowledged and contributes toward achieving health equity between indigenous and non-indigenous populations [18, 28]. We identified the culture-centered approach (CCA), community engagement/community-engaged research (CE/CEnR), systems thinking, and integrated knowledge translation (IKT) as areas that provide theoretical relevance to the context of implementation science in indigenous communities and conceptual fit with Kaupapa Māori (see Fig. 1).

### Kaupapa Māori

#### E tipu e rea mō ngā rā o tō ao (grow and branch forth for the days destined to you)

The starting line of this well-known whakataukī exhorts us to keep growing and learning. It provides a cultural foundation upon which to consider the value of other disciplinary traditions to Māori development. The Kaupapa Māori approach is reflective of the diverse *tikanga* (cultural protocols) and *mātauranga* (indigenous knowledge) that form the basis of both community action and indigenous research methodologies [29, 30]. Mātauranga Māori is the body of knowledge that underpins traditional Māori society and provides the basis of technological and philosophical skills of the community [31].

Indigenous scholars locate Kaupapa Māori in relation to critical theory with its notions of critique, resistance, struggle and emancipation [29, 32, 33]. The most prominent of these is the seminal writing of Graham Smith who places emphasis on the need for Kaupapa Māori principles to be in an active relationship with practice [30]. Colonial processes have undermined Māori social, economic and political structures resulting in a redistribution of power and resources in favour of Pākehā (European descent) settlers which is reflected in the current economic and socio-political inequities between Māori and Pākehā [6, 7, 34–36].

Three key features emerge from the Kaupapa Māori approach: a) addressing unequal power by transforming hegemonic structures and systems; b) reaffirming of the importance of tikanga and mātauranga in the development of relationships and program; and c) promoting greater community participation and control across the spectrum of program design, implementation, and evaluation [29, 30, 33]. The elements of our conceptual framework are consistent with these features.

### Culture-centered approach

#### Ko taku reo taku ohooho, ko taku reo taku mapihi maurea (my language is my awakening, my language is the window to my soul)

We use this whakataukī to emphasise the importance of bringing the voice of the culture into interventions. Kaupapa Māori stresses the importance of cultural protocols and knowledge to develop interventions. The CCA aims to transform “social structures surrounding health through dialogues with cultural members that create spaces for marginalized cultural voices” (p. 305) [37]. The CCA theorizes that domination from various social practices produces communicative erasure through rules, practices, and procedures that limit opportunities for participation and knowledge creation [38]. Centering the discourse with those people most affected empowers them to exercise their own agency; community members can make sense of and create localized health solutions framed by their everyday experiences [39–41]. The CCA closely aligns with Kaupapa Māori and the related notion of cultural safety developed by late-Māori scholar Irihapeti Ramsden. Cultural safety recognizes the importance of community members feeling safe and researchers and health professionals being reflexive of power and privilege and adjusting their behavior to enhance safety [42, 43]. Cultural safety and Kaupapa Māori also recognize tino rangatiratanga (self-determination) for constructing knowledge and defining problems and solutions [31, 43, 44]. The CCA asserts indigenous self-determination and ways of knowing, challenges power imbalances and transforms the way in which health interventions are developed and implemented by encouraging greater community voice and control at all levels [38, 40].

Three characteristics underpin cultural centeredness: community “voice” for problems and solutions, reflexivity, and structural transformation and resources. Voice for problems and solutions indicates that affected community members participate in defining the problem and also identifying relevant solutions [37, 38]. Lived experiences and participation are key guides for defining problems, and help to identify the relevant data needed to explain problems [45]. Inherent in participation is listening and shifting of the stance of the researcher to co-construct locally-derived understandings of health that respect local logics [46–49].

Reflexivity questions the unstated and taken-for-granted power and privilege from outsiders [37, 38]. Reflexivity continually interrogates the ways outsiders participate in the production of knowledge and the politics of knowledge construction and their efforts of collaboration that seek to undo these privileges. Reflexivity ensures that the research and intervention processes are co-constructed and localized through community participation.

Finally, the CCA underscores the role of structures and resources for the successful implementation of effective interventions to achieve health equity. Structure refers to health providers and systems that enable and constrain access to resources [41]. Structural transformation highlights the capacity of communities to interpret structures and to participate in processes of change on the basis of co-created meanings [37, 41]. Community capacities for decision-making and advocacy are developed through the culturally-centered processes during researcher/practitioner-community partnership [40, 50]. These processes equip communities with strategies to leverage relationships with external stakeholders to access resources for the community [51].

### Community engagement

#### He urunga tangata he urunga pahekeheke, he urunga oneone mau tonu (the support of others is unreliable, the support of your own is sure)

We use this whakataukī to illustrate the need to ground interventions in the community co-developed and supported by the status and self-determination of community members. CE and CEnR are advocated for by indigenous and non-indigenous researchers, community members, and public health practitioners working with indigenous communities as a method for improving health and achieving health equity [52–54]. CE is a process of collaborating with groups directly affected by a particular health issue or with groups who are working with those affected [55]. Although it overlaps with CCA in its interest in voice and power sharing, the unique focus of CE is partnership among community members and researchers/health professionals in developing interventions [56, 57]. Especially when guided by principles of shared power, mutual learning, and benefits for the community, CE enables the development of strong relationships that build the capacity of the communities and researchers [57, 58]. When following these principles, CE aligns with the focus on community participation advocated by Kaupapa Māori especially within the context of meeting obligations of the Treaty of Waitangi [24, 32, 59].

A number of recent systematic reviews and meta analyses have found compelling evidence supporting the positive impacts of CE on health outcomes and inequities [60–65]. Cyril and colleagues [66] completed a systematic review of the literature examining the impact of community engagement (CE) on health outcomes in disadvantaged populations. They identified 24 studies that met inclusion criteria and found that 88% of the studies had positive health outcomes. A meta-analytic review of 131 articles by O’Mara-Eves and colleagues [67] on randomised or non-randomised control trials of CE on a range of health outcomes for disadvantaged communities found that CE had positive impacts on health behaviour outcomes, increasing health consequences, health behaviour self-efficacy, and perceived social support.

A range of CE and CEnR approaches exist and there is a no consensus on an ideal approach. CE has been ranked into five categories, ranging from very limited community involvement to community ownership and management: outreach, consultation, involvement, shared leadership, and community-driven [55, 68]. One of the most popular approaches to CE is community-based participatory research (CBPR), accounting for 62% of CE approaches in a recent systematic review of studies addressing health disparities [66]. CBPR is popular in part because it moves beyond utilitarian engagement to a deeper value-based rationale for engagement [50, 69, 70]. CBPR involves partnership between researchers and community members/organizations in all phases of the research process and is guided by principles of action, social justice and power sharing [57, 58, 71, 72]. These principles are key reasons CBPR and other CE approaches are supported by indigenous scholars [18, 53, 54].

### Systems thinking

#### He tina ki runga, he tāmore ki raro (contentment above, firmly rooted below)

We use this whakataukī to highlight the importance of considering the implementation of interventions from a range of perspectives, levels and understandings. “Wicked problems,” such as health inequities, are characterised by high levels of complexity, uncertainty and conflict [73, 74]. These problems are not easily understood or tackled using a reductionist approach which breaks complex problems into smaller problems [75]. A systemic approach is needed to enable an appreciation of the ‘big picture’ and thus is consistent with Kaupapa Māori philosophy with its emphasis on holism and connection among levels, institutions, systems, and people [29, 30]. Several studies illustrate the links of systems thinking and Māori knowledge and health including multiple levels and systems from the individual to the spiritual and political [76, 77].

Systems thinking (especially system dynamics) has been applied to various public health issues including diabetes [78], obesity [79], and CVD [80, 81]. The use of systems thinking is relatively novel within public health, [82] and not without challenge given that it may run counter to some deeply ingrained assumptions and practices [83]. Systems thinking is “a general *conceptual* orientation concerned with the inter-relationships between parts and their relationships to a functioning whole, often understood within the context of an even greater whole” (italics in original, p. 539) [83]. System ideas, including sub-systems and the supra-system, mirror a socio-ecological understanding that situates the behaviour of individuals in relation to nested social, cultural, economic, political contexts [84].

Although systems approaches are characterised by a commitment to holism (i.e., the whole is more than the sum of the parts), the meaning and use of particular systems concepts varies depending on the approach [85]. The systems literature is vast and encompasses a wide range of hard, soft and critical traditions including general systems theory [86], system dynamics [87], soft systems [88], and critical systems thinking [85]. Take the concept of the ‘system’ as an example. For a hard systems thinker, a system is an entity that exists in the real world (e.g., health system). The literature on health care strengthening takes this perspective as does much of quality improvement literature [89, 90]. However, a soft systems thinker, cognisant of multiple perspectives and values would regard a system as a “particular way of organising our thoughts” (p. 2) [91]. For instance, Foote et al. [92] explored the ways in which primary and secondary care clinicians construct problems and solutions associated with a problematic hospital waiting list. Flood [93] helpfully distinguishes between systems and systemic thinking with the latter providing a set of constructs that can be utilised to address complexity.

System theorists see the diversity of systems approaches as a strength and contemporary systems thinking adopts a constructivist position that embraces both theoretical and methodological pluralism [94]. Although the systems approach is based on taking the ‘whole into account’, Midgley [85] points out that “there is no such thing as a genuinely comprehensive analysis, so the defining feature of systems thinking is the reflection of the boundaries of inclusion and exclusion” (pp. 7–8). A systemic understanding therefore requires attention to where boundaries are drawn. The act of boundary setting raises the question of who and what is included, and so enables or limits the opportunities for improvement [95]. For this reason, Daellenbach [96] notes that “an important aspect of systems thinking is the search for appropriate boundaries to the system … [and that] [b]oundary choices always involve some degree of arbitrariness and need to be challenged and justified by way of boundary critique” (p. 273). The process of exploring and critiquing the ‘givens’ of an intervention aligns with CCA’s concern to create spaces for subaltern voices and is often done in partnership with communities who are ‘affected but not involved’ as in CBPR processes [97]. In sum, systems thinking facilitates new framings, strategies and actions by considering what issues or viewpoints should be included in a systems analysis; how different perspectives are shaped by values and assumptions; and what interactions within and across institutional and organisational boundaries could produce better outcomes [98].

### Integrated knowledge translation

#### Toi te kupu, toi te mana, toi te whenua (hold fast to the language, the culture and the land)

We use this whakataukī to outline the process of taking research and working with community members to bring about positive outcomes for the community. The creation of new knowledge often does not, on its own, lead to widespread implementation or positive health outcomes [21]. Knowledge-translation processes offer the potential to build bridges between researchers/academics and communities to increase the potential for research to lead to improved health outcomes and health equity [99]. To understand and influence change in their practice settings, health care professionals and policy makers need to understand theories and frameworks that support knowledge translation [100]. The CIHR promotes IKT as a co-innovation approach involving knowledge users as equal partners alongside researchers to lead to research that is more relevant to and more useful to knowledge users [21]. The participatory nature of IKT is consistent with the collaborative investment approach of the NSC and Kaupapa Māori research practices [10, 99] although it is less attuned to the power differences expressed by the CCA. For example, the New Zealand Health Research Strategy recognizes the importance of partnership with Māori to achieve effective translation of research into policy and practice [99].

An important strategy in the context of indigenous communities is “the integration of relevant knowledge translation activities within the context in which the knowledge is to be applied” (p. 142) [101]. Health care environments are complex, so ensuring a fit between context and theory is important for the success of knowledge-translation initiatives [102]. Similarly, planning for knowledge translation is more likely to be successful in specific settings if an assessment of likely barriers and facilitators inform the choice of knowledge translation strategy [103]. Smylie et al. [101] suggest that due to the fundamental differences between Western scientific and indigenous knowledge systems, modification of current knowledge translation frameworks is necessary before they will be relevant in indigenous communities. In addition, knowledge translation methods for health research should be developed and evaluated specifically within the context of indigenous communities. The type of knowledge translation activity should be negotiated in conjunction with the relevant communities to decide what type of modification and level of support is required.

Gaps between evidence and decision-making exist in all levels of health care, including those of patients, health care professionals and policy-makers [104]. This is especially true for Māori communities which, through structural and resource constraints, have inequitable access to best available evidence [105]. The relevance and importance of IKT though such processes as uptake, implementation and dissemination, are vital to transition asynchronous research, practice, and public policy making processes into health gains for indigenous communities [106, 107].

Turning knowledge into action encompasses processes of knowledge creation and knowledge application. Knowledge creation and application have four levels, each with a growing level of engagement with indigenous communities: transfer, adoption, adaption and co-innovation [108]. Transfer is providing the knowledge or intervention to the community (doing the work for the community). Adoption provides a moderate level of support by providing basic knowledge about an intervention. Adaptation involves tailoring information to the needs of the knowledge user and includes feedback loops for adjusting the intervention. Co-innovation involves the co-design and co-implementation of knowledge and the intervention. Co-innovation reflects IKT and is also reflective of Kaupapa Māori and indigenous self-determination [31, 44].

## Applying the framework

A recent systematic review examined primary care interventions for Type 2 diabetes prevention in indigenous communities [23]. The review identified 13 articles with six having positive outcomes on diabetes and five having positive outcomes on blood pressure. The authors identified various factors including setting, location (rural or urban), intervention level, governance (shared, private, community), and study quality. The authors suggested that multifaceted interventions are most effective and need to involve some level of system change (clinical or health). We used the He Pikinga Waiora Implementation Framework to code the interventions in these studies to illustrate the potential post-hoc explanatory power of the framework. We offer this evidence as a preliminary indication rather than definitive proof of the framework given the small sample size.

Coding was completed in several stages. First, a coding scheme for each of the four elements was developed. Table 1 presents the final coding scheme and brief description of the variables and levels. The coding scheme was created in a two-step process involving initial definitions and application of the scheme to the studies by two coders and then a revision and reapplication by the coders. The coding was based only on the details provided in the 13 articles. Details were variable with four articles having limited information, four having moderate information, and five having in-depth information about intervention development. After the initial coding, interrater reliability was low (average ICC = .49). The first set of definitions relied on having specific details about intervention development and some manuscripts were lacking the relevant information. The study authors reviewed the definitions and suggested revisions to create more concrete categories that enabled consistent coding despite the variety of details about the interventions. In the second stage, the two coders independently reviewed the interventions without knowledge of the outcomes of the study. Final interrater reliability was strong (average ICC = .83). All disagreements at this stage were resolved through discussion by the two coders.

**Table 1:** Coding Scheme for Indigenous Implementation Framework

Variable/Definition	High	Medium	Low	Negative
Community voice: Community part of defining problem and identifying solutions. Community is group or groups that the intervention is focussed on.	Community involved in defining the problem and developing the solution	Community involved in either defining the problem or developing the solution. OR multiple communities involved but only one community involved in problem definition and solution development.	Community only informed or gives implicit approval but has no direct involvement in the definition of problem or solution development.	Intervention implemented in the face of significant community opposition
Reflexivity: Questioning the unstated and taken-for-granted power and privilege from which outsiders initiate contact with the community.	The implementation team explicitly states their reflexivity and identifies adjustments to the intervention as a result.	The implementation team identifies efforts to engage in reflexivity or states they were aware of it; adjustments to the intervention are unclear.	No evidence that the team was reflexive about its processes or, no changes made in response to team learning’s.	Victim blaming, unintended bias or overt racism in intervention design, implementation or evaluation.
Structural transformation and resources: Changing the nature of the system to better fit the community needs.	Significant structural transformation and resources which are sustainable over time.	Structural transformation and resources that are minimal or sustainable over the short term only.	Structural transformation and resources that are minimal and sustainable over the short term only.	Less resources available or lower quality resources as a result of the intervention compared with no intervention.
Community Engagement: The level of involvement, impact, trust and communication with community members	Strong community or bi-direction leadership. Decision making and communication is shared and a strong partnership is identified throughout the intervention process.	Communication is bidirectional and the community participates with the intervention team on the issues. Communication is two-way and there is cooperation to implement the intervention with a partnership becoming apparent.	Intervention is placed in the community with consultation. Communication primarily flows from intervention team to communition and the intervention team has ultimate control over the intervention and relavent communication.	N/A
Integrated Knowledge Translation: How the intervention is implemented with regard to the degree that the knowledge users are equal partners with the intervention team	There is a process of mutual or bi-directional learning established so that information is tailored to knowledge users needs.	Medium level support for knowledge user by intervention team for implementing the intervention. Intervention is not tailored to the knowledge user.	Minimal or no support for implementing intervention or outsiders implement the intervention for the knowledge users.	Knowledge users have major concerns about the intervention which they communicate to the intervention team, but they are not able to discuss their concerns with the intervention team.
System perspectives: The degree to which the team demonstrate recognition that there are multiple ways of viewing issues and solutions depending on worldviews, values and interests.	Intervention includes all three of the following: 1) multiple causes, 2) broad focus/multiple solutions; and 3) multiple perspectives, worldviews, and values of multiple actors in the system.	Intervention includes only on 2 of the 3 factors in high category.	Intervention includes 1 or none of the three factors in high category.	Intervention has a negative impact on other areas that will result in increasing the problem and issue would have been apparent had team explored multiple perspectives.
System relationships: Prioritises an understanding of relationships between variables/factors rather than taking a laundry list approach	Demonstrates strong understanding of the complex relationships between variables including feedback loops, time delays and multi-level effects.	Demonstrates moderate understanding of the complex relationships between variables including feedback loops, time delays and multi-level effects.	Limited or weak understanding of the complex relationships between variables including feedback loops, time delays and multi-level effects.	N/A
System levels: Takes different levels of analysis into account and provides clear rationale for the choices of levels.	The intervention targets change at the macro, meso and micro levels, and provides sufficient rationale and context for each level.	The intervention targets change at the three levels but does not provide rationale and context for each level. Or, the intervention targets two levels and provides rationale and context.	The intervention targets change at two levels or less without providing rationale and context.	N/A

Coding results are presented in Table 2 along with the results of two common outcomes across the studies: improved diabetes outcomes (HbA1c or amputation incidence) and improved blood pressure (either diastolic or systolic). Both outcomes were coded as changed or unchanged using significance values provided in the articles. In order to have an equal weighting among the four constructs in the scheme, we recoded the three culture-centeredness variables into a single variable, and the three systems variables into a single variable, based on most common rank or average of ranks if a common rank was not obtained.

**Table 2:** Coding Results and Outcomes

Study	Culture-Centeredness			Community Engagement	Integrated Knowledge Translation	Systems Thinking			Outcomes	
	Voice	Reflexivity	Transformation & Resources	Community Engagement	Integrated Knowledge Translation	Systems Perspective	Systems Relation	Systems Level	Diabetes Outcome	Blood Pressure
Bailie et al. 2004 [114]	Low	Low	Medium	Low	Medium	Low	Low	Low	Unchanged	Unchanged
Bailie et al. 2007 [115]	Medium	Medium	Medium	Low	Medium	Medium	High	High	Changed	Unchanged
Roubideaux et al. 2008 [116]	Low	Low	Medium	Low	Low	Low	Medium	Medium	Unchanged	Unchanged
Wilson et al. 2005 [117]	Medium	Medium	High	Medium	Medium	High	Medium	High	Changed	Changed
Kenealy et al. 2010 [118]	Medium	Medium	High	Medium	Medium	Medium	Medium	Medium	Changed	Unchanged
Smith et al. 2011 [119]	Low	Low	High	Low	Low	Low	Low	Medium	Unchanged	Changed
Simmons 2003 [120]	Medium	Low	Medium	Medium	High	Medium	Medium	Medium	Changed	Changed
Schraer et al. 2003 [121]	Medium	Medium	High	Medium	Medium	Medium	Medium	Medium	Changed	N/A
Ramesh et al. 2008 [122]	Medium	Low	High	Medium	Medium	Medium	Medium	Medium	Changed	Changed
Virani et al. 2006 [123]	Low	Low	Medium	Low	Medium	Medium	Medium	Medium	Unchanged	Unchanged
McDermott et al. 2001 [124]	Low	Low	Medium	Low	Medium	Medium	Medium	Medium	Unchanged	Unchanged
Tobe et al. 2006 [125]	Low	Low	Medium	Low	Medium	Medium	Low	Medium	Unchanged	Changed
Ralph-Campbell et al. 2006 [126]	Low	Low	High	Low	Medium	Medium	Medium	Medium	Unchanged	Unchanged

Cross-tabulations were calculated using each of the outcome variables and the four elements of the Framework. Table 3 presents the frequencies and the chi-square value and significance for the associations. Culture-centeredness and CE had a significant association with diabetes outcome; system thinking and IKT had a potential association with diabetes outcome (*p* < .15). For example, the cells for community engagement and diabetes outcome illustrate that five studies with a changed diabetes outcome had a medium-level of engagement and one study has low engagement. In contrast, all seven studies with an unchanged diabetes outcome had a low-level of engagement. Thus, higher levels of community engagement is associated with an improvement in diabetes outcome, χ^2^ (2, *N* = 13) = 9.48, *p* = .009. CE had an association at the .10 level and culture-centeredness at a .20 level with blood pressure. The liberal *p* value is used for these analysis given the small sample size. In conclusion, the framework appears to distinguish between changed/unchanged diabetes and blood pressure outcomes for primary care interventions in indigenous communities and provides a potential post-hoc explanation to why some interventions had better outcomes than others.

**Table 3:** Frequency Counts and Associations

Framework Element	Ranking (out of 13)	Outcomes					
		Diabetes (*n* = 13)			Blood Pressure (*n* = 12)		
		Changed	Unchanged	Change vs. Unchanged χ^2^	Changed	Unchanged	Change vs. Unchanged χ^2^
Culture-Centeredness	High (*n* = 0)	0	0	6.96 (*p* = .008)	0	0	1.66 (*p* = .20)
	Med (*n* = 8)	6	2		4	3	
	Low (*n* = 5)	0	5		1	4	
Community Engagement	High (*n* = 0)	0	0	9.48 (*p* = .009)	0	0	2.74 (*p* = .098)
	Med (*n* = 5)	5	0		3	1	
	Low (*n* = 8)	1	7		2	6	
Integrated Knowledge Transfer	High (*n* = 1)	1	0	2.94 (*p* = .23)	1	0	1.71 (*p* = .42)
	Med (*n* = 10)	5	5		3	6	
	Low (*n* = 2)	0	2		1	1	
Systems Thinking	High (*n* = 2)	2	0	4.06 (*p* = .13)	1	1	0.17 (*p* = .92)
	Med (*n* = 9)	4	5		3	5	
	Low (*n* = 2)	0	2		1	1	

## Implications and conclusions

The He Pikinga Waiora Implementation Framework provides a theoretically-sound foundation for enhancing the implementation of health interventions for Māori and other indigenous communities because it centers indigenous knowledge and self-determination. The four elements are wrapped around a center grounded in indigenous critical theory (i.e., Kaupapa Māori) and each element is consistent with, and supportive of, indigenous knowledge creation and use. The four elements are related and yet each adds a distinct component to the framework. Culture-centeredness recognizes the importance of local perspectives, reflexivity, and also the importance of using these elements to leverage resources and create structural change [37, 40]. These elements support indigenous perspectives to define and solve problems which is consistent with Kaupapa Māori.

CE and CEnR emphasize bidirectional learning, power sharing, and collaborative partnerships [57, 66]. Consistent with Kaupapa Māori and culture-centeredness, CE recognizes the importance of voice and community agency and the need to share power. However, CE is the means to which culture-centeredness occurs. CE and CEnR involve clinicians, researchers, and policy makers working with communities rather than introducing top-down interventions. This approach enables co-created interventions to enhance implementation effectiveness and sustainability [52, 71]. Further, a CBPR approach guided by a conceptual model has strong support for outcomes related to health equity [18, 57, 109, 110].

Systems thinking recognizes the importance of holistic and multilevel thinking to address complex public health problems [85]. It avoids a reductionist approach to interventions, which is common in efficacy trials. The emphasis on holism fits well with Kaupapa Māori [31]. Systems thinking provides a unique theoretical element as it looks at the dynamics and connections among elements. Recent research demonstrates the connection between CEnR and systems theory for addressing health inequities [75, 111].

IKT recommends co-creation and co-innovation involving knowledge users as equal partners alongside researchers, policy makers, and practitioners to develop perspectives that are relevant and useful for end users [21]. Co-innovation rather than simply transferring knowledge from researcher to end user is reflective of Kaupapa Māori as it is at least co-driven by Māori. This element also adds a unique component to the model in how knowledge about an intervention is constructed to fit for end users through community feedback. It is similar to recognition of voice in culture-centeredness and yet distinct because it recognizes different levels in how knowledge is shared with others.

Individually, these four elements have an evidence-base demonstrating improved implementation effectiveness for indigenous and other communities experiencing health inequities. Collectively, the elements should provide a more complete picture of implementation effectiveness. The framework suggests participatory approaches of CE and culture-centeredness ensure self-determination and indigenous perspectives are present and also that design attributes are consistent with the perspectives of the various communities being served. The systems thinking reflects a complex understanding of the chronic disease workforce and clinical care pathways and enables a coordinated approach to the intervention. IKT supports the communication of new evidence across the system in a manner appropriate for the community and professional setting to improve the quality of services and outcomes for communities. Further, the framework is consistent with key practices identified in recent reviews of implementation science research [112, 113]. Gibson and colleagues [112] identified key themes from 23 studies of enablers and barriers to implementing primary healthcare interventions in indigenous communities including intervention design created in partnership with the community, integration of intervention with organizations along with clear clinical care pathways, and culturally safe access to services.

Our coding of primary care interventions for prevention of diabetes tentatively shows that elements of the framework have potentially differential contributions to outcomes. CE was important for blood pressure outcomes, and culture centeredness, CE, and systems thinking were important for diabetes outcomes. Our framework provides a different read than the meta-analysis study authors offered [23]. The authors found a multifaceted systems perspective was a key feature of successful interventions. We found that CE, culture-centeredness, and systems thinking also have explanatory power with a potential influence for IKT. We reiterate that this current study is indicative and not definitive and yet coupled with the extant literature provides support for the usefulness of the collective framework. In addition, this study has a limitation in that we coded information about interventions from the published articles and not the interventions themselves. Although it is likely that authors highlighted key elements on their intervention development in the articles, limitations in publication space sometimes restricts the presentation of full details on interventions.

Further research hopefully will provider stronger evidence of the usefulness and validity of the framework particularly with Māori end-users. We have begun additional research including co-designing health interventions following this framework with two community organisations and also interviewing policy makers, researchers, and practitioners about using the framework as a platform for Māori implementation science. There will also need to be further research to understand the differential contribution of each of the four elements to health outcomes.

The framework also has implications for funders, researchers, and community and public health organizations. Specifically, this framework can be used as a planning tool to guide successful development and implementation of interventions for communities experiencing the burden of health inequities. Funders can use the framework to assess likelihood of effectiveness for proposed interventions or perhaps use this framework to rate applications that address these four elements (e.g., “bonus points” beyond established criteria). Community organizations and indigenous tribal leaders can use these elements to help decide whether to work with researchers or policy makers proposing a specific intervention. These organizations can ask the potential collaborators how they will foster each of the elements in the framework and whether they will work in partnership.

In conclusion, improving health outcomes for Māori and other indigenous communities is a goal shared by governments, agencies, and communities themselves. Despite improvements in health for most populations, continuing health inequities are prominent and of great concern. This study proposes the He Pikinga Waiora Implementation Framework for intervention effectiveness particularly for non-communicable diseases such as diabetes, CVD, and obesity. Our work suggests that centering indigenous perspectives through ensuring community voice, collaborative partnership, systems thinking, and the collaborative creation of knowledge represents a promising approach for improving health and achieving health equity. The Healthier Lives Challenge NSC is implementing the Framework in part, and over the next decade we will have further evidence of the impact of such a framework on health and equity.
